# *N*-acetylcysteine negatively regulates Notch3 and its malignant signaling

**DOI:** 10.18632/oncotarget.8806

**Published:** 2016-04-18

**Authors:** Xiong Zhang, Ya-Nan Wang, Juan-Juan Zhu, Xue-Xia Liu, Hui You, Mei-Ying Gong, Ming Zou, Wen-Hsing Cheng, Jian-Hong Zhu

**Affiliations:** ^1^ Department of Preventive Medicine, Wenzhou Medical University, Wenzhou, Zhejiang 325035, China; ^2^ Department of Geriatrics and Neurology, The Second Affiliated Hospital, Wenzhou Medical University, Wenzhou, Zhejiang 325035, China; ^3^ Institute of Nutrition and Diseases, Wenzhou Medical University, Wenzhou, Zhejiang 325035, China; ^4^ Key Laboratory of Watershed Science and Health of Zhejiang Province, Wenzhou Medical University, Wenzhou, Zhejiang 325035, China; ^5^ Department of Food Science, Nutrition and Health Promotion, Mississippi State University, Mississippi State, Mississippi 39762, USA

**Keywords:** N-acetylcysteine, Notch3, ROS, lysosome, malignancy

## Abstract

Notch3 receptor is expressed in a variety of cancers and the excised active intracellular domain (N3ICD) initiates its signaling cascade. *N*-acetylcysteine (NAC) as an antioxidant has been implicated in cancer prevention and therapy. In this study, we demonstrated a negative regulation of Notch3 by NAC in cancer cells. HeLa cells treated with NAC exhibited a time- and concentration-dependent decrease in Notch3 levels and its downstream effectors Hes1 and HRT1 in a manner independent of f-secretase or glutathione. In contrast, NAC did not affect protein levels of Notch1, the full length Notch3 precursor, or ectopically expressed N3ICD. Although SOD, catalase and NAC suppressed reactive oxygen species in HeLa cells, the first two antioxidants did not impact on Notch3 levels. While the mRNA expression of Notch3 was not altered by NAC, functional inhibition of lysosome, but not proteasome, blocked the NAC-dependent reduction of Notch3 levels. Furthermore, results from Notch3 silencing and N3ICD overexpression demonstrated that NAC prevented malignant phenotypes through down-regulation of Notch3 protein in multiple cancer cells. In summary, NAC reduces Notch3 levels through lysosome-dependent protein degradation, thereby negatively regulates Notch3 malignant signaling in cancer cells. These results implicate a novel NAC treatment in sensitizing Notch3-expressing tumors.

## INTRODUCTION

The evolutionarily conserved Notch signaling is involved in many important processes, including cell fate determination, differentiation, and proliferation [1–3]. Upon ligand binding, the canonical activation of Notch signaling starts with the sequential cleavages of Notch receptor by tumor necrosis factor-α-converting enzyme and γ-secretase. An active intracellular domain is then released and translocates to the nucleus, where it associates with the DNA-binding protein CSL/RBP-Jκ for transcriptional activation of multiple downstream targets, including members of the Hes and HRT family [4, 5]. Notch3 is mainly expressed in vascular smooth muscle cells and facilitates cell orientation during arterial development [6, 7]. Pathologically, Notch3 expresses in cancer cells of various origins, including breast, ovary and colon, and plays a role in the regulation of tumor progression [6, 8–11].

*N*-acetylcysteine (NAC), a precursor of *L*-cysteine and reduced glutathione (GSH), is a widely-used antioxidant against reactive oxygen species (ROS) [12] and inhibits redox-sensitive signaling such as NFκB and MAPK pathways [13–17]. In addition to its applications in cardiovascular injury, pulmonary diseases and drug toxicity [18–20], NAC has been proposed as a chemopreventive agent, either stand-alone or as an adjuvant [21–24]. Nonetheless, a recent study employing a genetically engineered mouse model of cancer indicates that combined dietary supplementation of NAC and vitamin E increases cancer burden and mortality [25]. Chandel and Tuveson [26] have proposed that failure of chemoprevention by dietary antioxidants may be attributed to their limited access to the local, tumor-promoting ROS but not to the distant, oncogene-suppressive ROS.

Although NAC is best known as an antioxidant, we have shown in this study that NAC effectively suppresses Notch3 receptor levels through ROS-independent and lysosome-mediated pathways, and thereby inhibits Notch3 malignant signaling. Such results shall be of clinical significance for a new application of NAC in cancer treatment.

## RESULTS

### NAC down-regulates Notch3 receptor and its downstream signaling in HeLa cells

**Figure 1:**
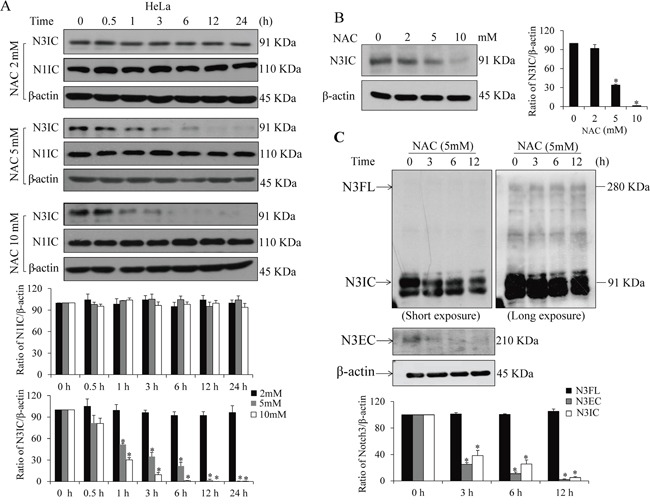
NAC decreases protein levels of Notch3, but not Notch1 **A.** Time- and dose-dependent inhibition of NAC on the intracellular domain of Notch3 (N3IC), but not Notch1 (N1IC). HeLa cells were treated with NAC (2-10 mM) for 0-24 h. **B.** Dose-dependent inhibition by NAC (0-10 mM, 6 h) on the protein expression of N3IC in HeLa cells. **C.** NAC treatment (5 mM, 0-12 h) reduces protein levels of N3IC and extracellular domain of Notch 3 (N3EC) but not full length Notch 3 precursor (N3FL) in HeLa cells. Densitometry quantifications of the protein bands were shown after normalization with their respective β-actin levels. Data are presented as means ± SE, n=3. *, *p* < 0.05 compared with their respective non-treated group.

**Figure 2:**
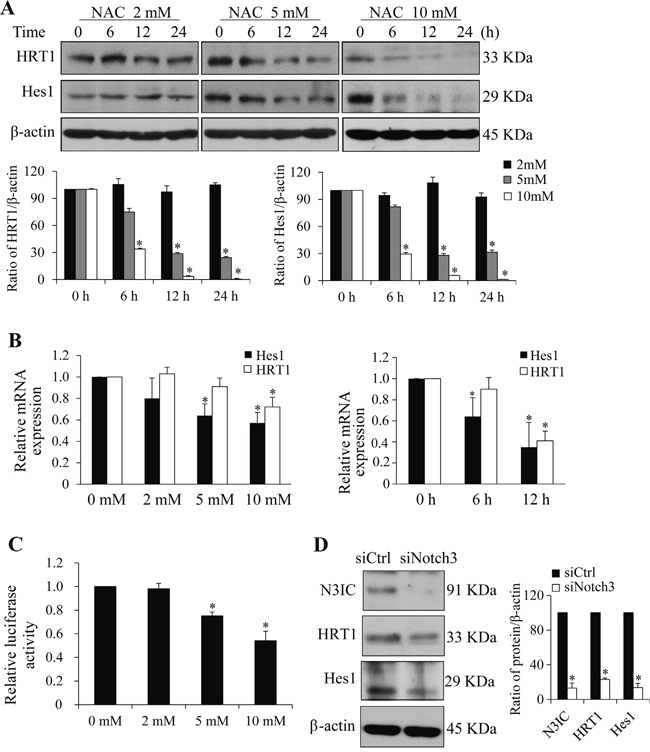
NAC inhibits Notch3 downstream signaling **A.** NAC treatment (2-10 mM, 0-24 h) decreases Hes1 and HRT1 protein levels in HeLa cells. **B.** NAC treatment (0-10 mM for 6 h or 5 mM for 0-12 h) decreases Hes1 and HRT1 mRNA expression in HeLa cells. The mRNA expression of NAC-treated cells was normalized to that of non-treated cells whose value was set as 1. **C.** NAC treatment (0-10 mM, 12 h) inhibits Hes1 reporter activity in HeLa cells. The luciferase activity in NAC-treated cells was normalized to that of non-treated cells whose value was set as 1. **D.** Notch3 siRNA knockdown reduces Hes1 and HRT1 levels in HeLa cells. Protein levels were determined 2 days after siRNA transfection. siCtrl, scramble siRNA; siNotch3, Notch3 siRNA. Protein densitometry quantifications were shown after normalization with β-actin levels. Data are presented as means ± SE, n=3-4. *, *p* < 0.05 compared with their respective non-treated group.

Mature Notch3 is a transmembrane dimer composed of an extracellular domain (N3EC) and an intracellular domain (N3IC), which are non-covalently bound after S1 cleavage of the full-length Notch3 precursor (N3FL) in Golgi complex [4, 5]. Treatment of HeLa cells with NAC time- and dose-dependently suppressed protein levels of N3IC (Figure 1A&1B) but not N1IC (Figure 1A). Further analyses showed that the protein levels of N3IC and N3EC, but not N3FL, were decreased after NAC treatment in HeLa cells in a time-dependent manner (Figure 1C). Next, N3IC transactivation targets were analyzed. Treatments of HeLa cells with NAC (2, 5 or 10 mM) resulted in dose- and time-dependent decreases in Hes1 and HRT1 protein (Figure 2A) and mRNA (Figure 2B) levels. Consistent with these observations, results from dual-luciferase reporter assays showed the Hes1 reporter activity was decreased in a dose-dependent manner following NAC treatment (Figure 2C). Furthermore, the protein levels of Hes1 and HRT1 were diminished upon siRNA knockdown of *Notch3* in HeLa cells (Figure 2D), confirming that these two genes are responsive downstream targets of Notch3 signaling in HeLa cells.

### NAC leads to Notch3 degradation through a lysosome-dependent pathway

**Figure 3:**
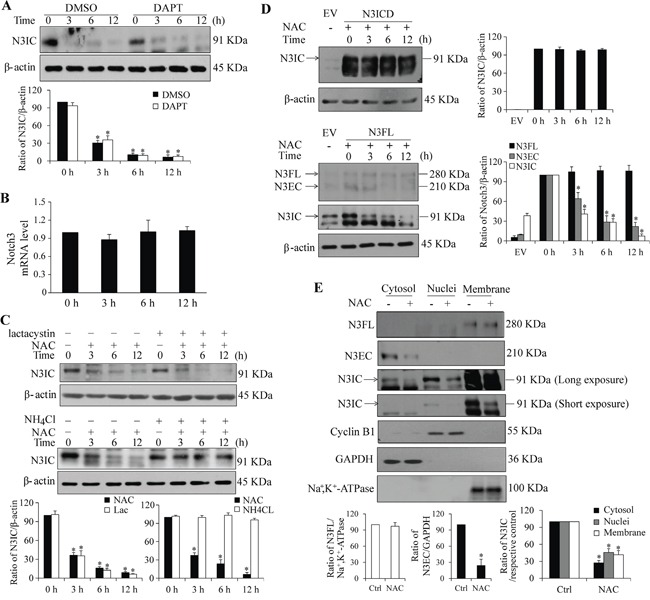
The NAC-induced decrease in Notch3 levels depends on lysosome-, but not proteasome-mediated proteolysis **A.** Pre-treatment with a γ-secretase inhibitor, DAPT (20 μM, 30 min), had no effect on NAC-induced (5 mM, 0-12 h) decrease in N3IC protein expression in HeLa cells. **B.** NAC treatment (5 mM, 0-12 h) did not affect Notch3 mRNA expression in HeLa cells. **C.** Pre-treatment with NH_4_Cl (25 mM, 1 h), but not lactacystin (10 μM, 30 min), reversed NAC-induced (5 mM, 0-12 h) decrease of N3IC protein levels in HeLa cells. **D.** NAC treatment did not affect levels of exogenously expressed Notch3 active intracellular domain (N3ICD). HeLa cells were transfected with vectors expressing N3ICD or N3FL for 24 h, followed by treatment with NAC (5 mM, 0-12 h). **E.** Subcellular analysis of Notch3 protein levels following NAC treatment (5 mM, 6 h) in HeLa cells. Protein levels of N3FL, N3EC and N3IC in cytosolic, nuclear and membrane fractions were determined. Successful fractionation was evidenced by using the marker proteins GAPDH, cyclin B1, and Na^+^, K^+^-ATPase. N3FL, N3EC and N3IC denoted Notch3 full length, extracellular domain and intracellular domain, respectively. Protein densitometry quantifications were shown after normalization with β-actin (A, C, D) or their respective cellular compartment markers (E). Data are presented as means ± SE, n=3-4. *, *p* < 0.05 compared with their respective non-treated group.

To understand the mechanisms underlying NAC-mediated Notch3 down-regulation, the canonical Notch processing by γ-secretase cleavage was firstly analyzed. Results from Western analysis showed that inhibition of γ-secretase activity by DAPT had no significant impact on the NAC-induced reduction in N3IC levels (Figure 3A). The mRNA level of Notch3 was not altered following NAC treatment (Figure 3B), indicating that the modulation occurred at protein level. Intracellular protein degradation may be achieved through lysosome-mediated proteolysis or ubiquitin-mediated proteasome process [27]. To test these possibilities, HeLa cells were pre-treated with lactacystin (a proteasome inhibitor) or NH_4_Cl (a lysosome inhibitor). The results showed that NH_4_Cl, but not lactacystin, blocked the NAC-induced decrease of N3IC protein levels (Figure 3C). Interestingly, NAC did not decrease the level of ectopically expressed active intracellular domain (N3ICD) when cells were transfected with a vector expressing N3ICD (Figure 3D, upper panel). In contrast, when cells were transfected with a N3FL vector, the levels of N3EC and N3IC, but not that of N3FL, were reduced following NAC treatment (Figure 3D, lower panel). Results from subcellular analyses of Notch3 proteins (Figure 3E) showed that N3FL was detected only in the membrane fraction and remained unchanged following NAC treatment. N3EC was detected only in the cytosolic fraction, most likely due to its dissociation from the dimerization at the condition of extraction and then shedding off from the membrane as N3EC is non-covalently bound with N3IC which is the one containing the transmembrane domain. N3IC was observed in all three fractions, with membrane being the main compartment. Subcellular levels of N3EC and N3IC proteins were decreased following NAC treatment (Figure 3E).

### NAC-mediated Notch3 down-regulation is ROS-independent

**Figure 4:**
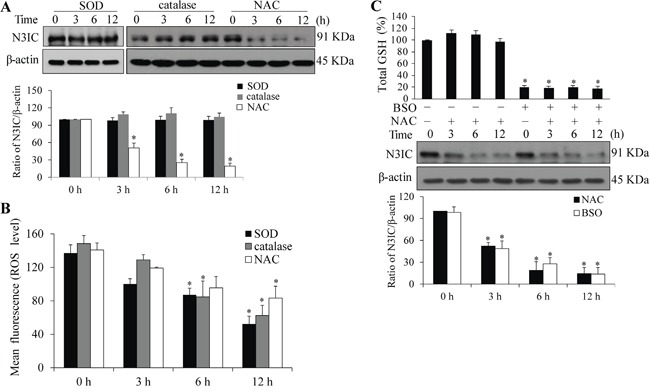
NAC-mediated Notch3 down-regulation is independent of ROS **A.** SOD or catalase treatment does not affect N3IC protein levels. HeLa cells were treated with SOD (1200 U/ml), catalase (1200 U/ml) or NAC (5 mM) for 0, 3, 6 or 12 h. **B.** The ROS levels following the treatments as described above. **C.** GSH depletion does not alleviate NAC-induced decrease of N3IC protein levels. HeLa cells were pretreated with BSO (100 μM, 24 h), followed by treatment with NAC (5 mM, 0-12 h). Total cellular GSH were measured and the values were normalized to that of the non-treated cells which was set as 100%. Protein densitometry quantifications were shown after normalization with β-actin levels. Data are presented as means ± SE, n=3. *, *p* < 0.05 compared with their respective non-treated group.

To determine whether the NAC-mediated Notch3 down-regulation is attributed to its antioxidant function, additional antioxidants, SOD and catalase enzymes, were used to test this possibility. Treatment of HeLa cells with SOD or catalase did not lead to the reduction of N3IC protein level as observed by NAC treatment (Figure 4A, upper panel), although intracellular ROS levels were decreased at a comparable level between these three antioxidants (Figure 4B). Since NAC is a precursor of GSH, we thenceforward determined whether GSH confers the effect of NAC on Notch3. Cellular GSH level was increased with NAC addition but depleted by BSO, a glutathione-synthesis inhibitor (Figure 4C, upper panel). However, the NAC-induced decrease of N3IC protein level was independent of BSO treatment (Figure 4C, lower panel).

### NAC alleviates Notch3-mediated malignant phenotypes

**Figure 5:**
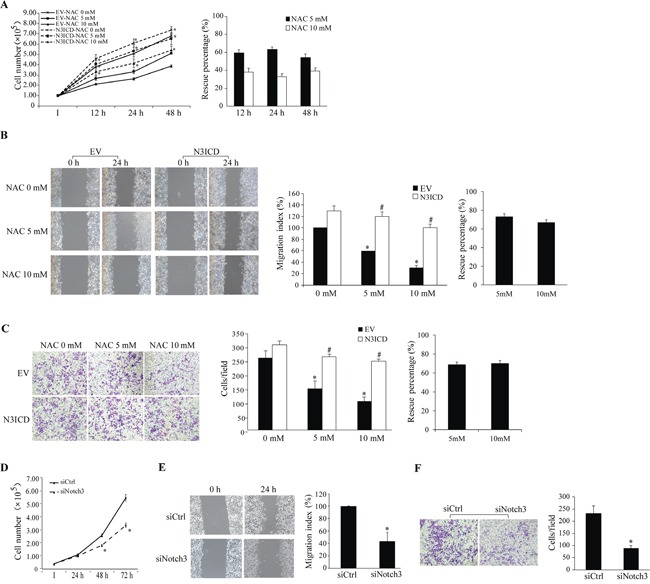
Impact of Notch3 and NAC on HeLa cell malignancy N3ICD overexpression rescues NAC-induced inhibition of proliferation (A), migration (B), and invasion (C). **A.** Numbers of EV- and N3ICD-transfected cells were counted at 12-48 h after NAC treatment (0-10 mM, left panel). *, *p* < 0.05 compared with the EV-transfected cells within the same treatment and time point. **B.** Results of the wound healing assay (left panels) were expressed as the migration index (the distance migrated relative to the initial scraped gap) and that of EV-transfected cells without NAC treatment was set as 100% (middle panel). **C.** Cells per field on the insert membrane were imaged (left panels) and counted (middle panel). B and C: *, *p* < 0.05 compared with no NAC treatment; #, *p* < 0.05 compared with the EV-transfected cells within the same treatment. Percent rescue (A-C, right panels) after N3ICD expression was calculated by dividing the net change after NAC treatment in N3ICD-transfected cells by that in EV-transfected cells. Notch3 siRNA knockdown inhibits cell proliferation **D.**, migration **E.**, and invasion **F.** as assessed by the same approaches described above. Representative images for migration and invasion were shown. *, *p* < 0.05 compared with the siCtrl-transfected cells. All data are presented as mean ±SE, n=3. I, the initial seeded cell number. EV, empty vector; N3ICD, Notch3 active intracellular domain; siCtrl, scrambled siRNA; siNotch3, Notch3 siRNA.

HeLa and HCC1937 cells were used to assessed the effects of NAC-Notch3 on cell proliferation, migration and invasion, in which no apparent difference was observed among naive cells, lipofectamine-treated, and empty vector-transfected cells (Supplementary Figure S1). While treatment with NAC (5 or 10 mM) led to significant inhibition in cell proliferation (Figure 5A), migration (Figure 5B) and invasion (Figure 5C) in EV-transfected HeLa cells, exogenous expression of N3ICD reversed such inhibitory effects of NAC. To better evaluate the actual impact of N3ICD on cell malignancy, graphs of net percent changes were presented (Figure 5A–5C, right panels). Expression of exogenous N3ICD rescued the inhibition of NAC at 5 and 10 mM, respectively, on 1) cell proliferation by 59.6±3.4 and 38.3±4.2% at 12 h, 63.3±2.3 and 32.9±3.4% at 24 h, and 54.4±3.6 and 38.9±3.4% at 48 h (Figure 5A), 2) cell migration by 74.1±3.3 and 67.0±2.9 % at 24 h (Figure 5B), 3) cell invasion by 68.7±2.9 and 70.0±3.1% at 24 h (Figure 5C). Furthermore, siRNA knockdown of Notch3 in HeLa cells resulted in blunted proliferation, migration and invasion (Figure 5D–5F), suggesting that HeLa cell malignancy is Notch3-dependent.

**Figure 6:**
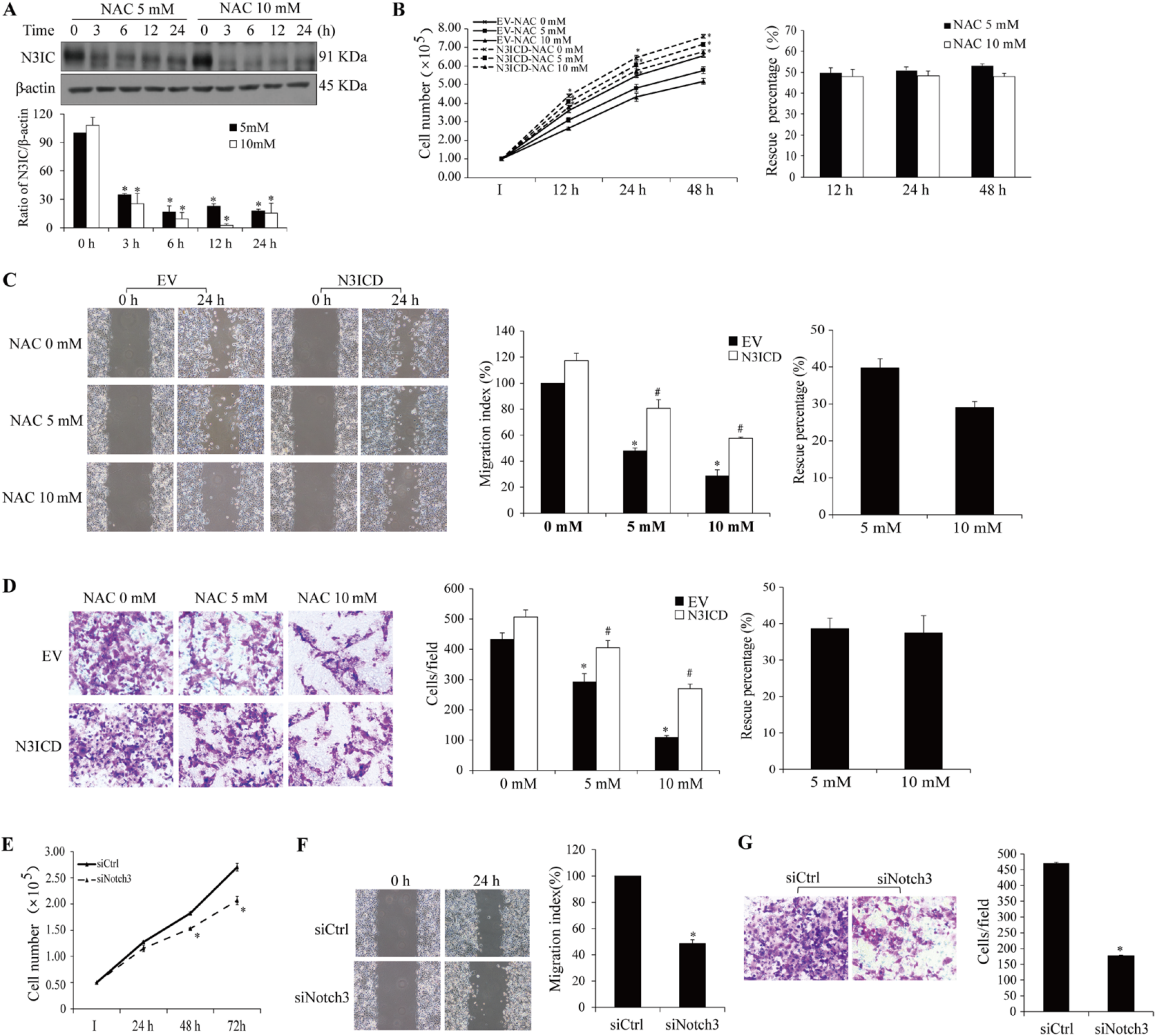
Impact of Notch3 and NAC on HCC1937 cell malignancy **A.** NAC treatment (5 and 10 mM, 0-24 h) decreases N3IC protein levels in HCC1937 cells. Expression of exogenous N3ICD rescues NAC-induced inhibition of proliferation **B.**, migration **C.**, and invasion **D.**, and Notch3 siRNA knockdown inhibits proliferation **E.**, migration **F.**, and invasion **G.** in HCC1937 cells. Quantifications, sample size, statistics, and abbreviations for protein levels, proliferation, migration, and invasion assays were as described in Figure 1A&5 legends.

Additional cancer cells were employed to explore whether the inhibitory effect of NAC on Notch3-meditated malignancy is cell-specific or a general pathway. Analyses in HCC1937 breast cancer cells showed similar outcomes of NAC inhibition of Notch3-mediated malignancy as those in HeLa cells, including NAC-induced decrease in N3IC protein level (Figure 6A) and rescue of NAC-inhibited proliferation, migration and invasion by N3ICD (Figure 6B–6D). The percent rescue was averagely 51.1±0.8 and 48.1±0.1% for proliferation (Figure 6B), 39.8±2.5 and 29.0±1.7% for migration (Figure 6C), and 38.6±2.9 and 37.5±4.7% for invasion (Figure 6D) following NAC treatment at 5 and 10 mM, respectively. Similarly, siRNA knockdown of *Notch3* attenuated HCC1937 cell proliferation, migration and invasion (Figure 6E–6G).

### NAC causes Notch3 degradation and growth inhibition in multiple cancer cells

**Figure 7:**
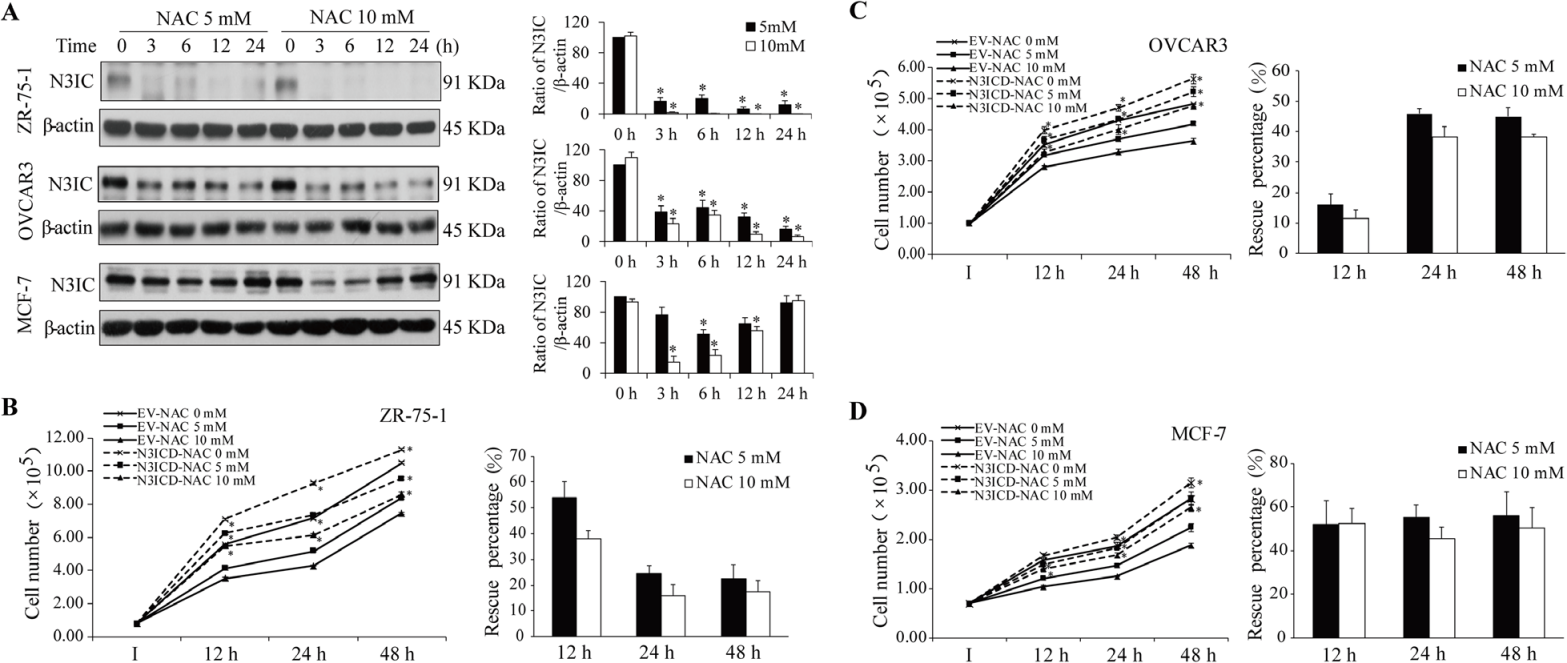
Analysis of the NAC-Notch3 pathway in three other cancer cells **A.** NAC treatment (5 and 10 mM, 0-24 h) decreases N3IC protein levels in ZR-75-1, OVCAR3 and MCF-7 cells. **B-D.** Expression of exogenous N3ICD rescues NAC-induced inhibition of proliferation in ZR-75-1 (B), OVCAR3 (C), and MCF-7 (D) cells. Quantifications, sample size, statistics, and abbreviations for protein levels and proliferation assays were as described in Figure 1A&5 legends.

In addition, three potential Notch3-dependent cancer cell lines ZR-75-1, OVCAR-3 and MCF-7 [6, 10, 28, 29] were used to evaluate the effects of NAC on Notch3 protein levels and cell proliferation. Similarly, N3IC protein levels were decreased upon NAC treatment (5 and 10 mM, 0-24 h) in these three cell lines (Figure 7A). Interestingly, the NAC-induced decrease in N3IC protein levels was gradually recovered in MCF-7 cells, presumably attributed to a high efficiency in regenerating Notch3 receptor in this cell line. While NAC treatment does-dependently inhibited the proliferation of ZR-75-1 (Figure 7B), OVCAR3 (Figure 7C), and MCF-7 (Figure 7D) cells, expression of exogenous N3ICD attenuated such inhibitory effect of NAC by averagely 33.6±8.3 and 23.8±5.8% in ZR-75-1 cells (Figure 7B), 36.0±7.8 and 29.4±7.2% in OVCAR3 cells (Figure 7C), 54.5±1.1 and 49.6±1.6% in MCF-7 cells (Figure 7D), at 5 and 10 mM, respectively.

## DISCUSSION

Emerging evidence have suggested that Notch3 is expressed in a number of cancerous tissues and plays an oncogenic role [6, 8–11]. NAC is a commonly used antioxidant either in clinic or basic research. In this study, we have disclosed a novel regulation of Notch3 receptor by NAC. Through a ROS-independent, lysosome-mediated pathway, NAC counteracts Notch3 malignant signaling in cancer cells.

The canonical process of Notch activation is dispensable in the NAC-induced Notch3 down-regulation as this novel pathway is independent of γ-secretase activity. As revealed by the analyses of Notch3 endogenous expression, N3FL exogenous expression and their subcellular expression, NAC unanimously induces a reduction in N3IC and N3EC levels while the levels of N3FL remain unchanged, suggesting that the S1-cleaved mature Notch3, but not the full-length Notch3 precursor, is targeted by NAC. This notion is further supported by our mRNA analyses showing that Notch3 is not altered by NAC transcriptionally. Furthermore, NAC does not affect the protein levels of ectopically expressed N3ICD. These results together indicate that the regulation of NAC on Notch3 occurs at protein level and NAC targets Notch3 at the interaction face of its intracellular and extracellular domains, i.e., the non-covalent binding region. Interestingly, the expression of Notch1 is not affected by NAC. Sequence differences between Notch1 and Notch3 exist along the heterodimerization region of the two domains [30, 31], which share only 41% homology (Supplementary Figure S2). NAC has been suggested to reduce protein disulfides in favor of thioredoxin in some cases, and to bind sulfenic acid derivatives in proteins [32]. Therefore, it is proposed that NAC modifies the non-covalent binding region of Notch3, decreasing protein stability and leading to protein degradation. Indeed, a lysosome-mediated proteolysis, but not the ubiquitin-proteasome pathway, is found to be involved in the degradation of Notch3 by NAC. A critical role for lysosomes in Notch3 degradation is also affirmed in the auto-degradation of Notch3 fragments [33].

Being effective antioxidants, NAC, SOD and catalase are functionally comparable and often comparably used in blocking ROS-generated cellular signaling [24, 34–37]. Although incubation of cells with SOD and catalase indeed lead to similar decrease of intracellular ROS as compared to NAC, these two antioxidants surprisingly do not affect N3IC protein levels. Furthermore, inhibition of GSH synthesis, which is boosted by NAC treatment, does not affect the NAC-induced decrease in N3IC protein level. These results suggest that NAC mediates Notch3 protein level through a ROS-independent mechanism.

Hes1 and HRT1 mRNA and protein levels are markedly reduced following NAC treatment. Since these two genes are targeted by both Notch1 and Notch3 but only the Notch3 expression is inhibited by NAC, one possibility is that Notch1 may compensate for Notch signaling when Notch3 is down-regulated in the presence of NAC. However, siRNA knockout of Notch3 leads to significant decreases in Hes1 and HRT1 levels, indicating that Notch signaling in HeLa and HCC1937 cells is indeed dependent on Notch3, if not exclusively. In agreement with most reports [21–24], NAC administration attenuates cancer cell proliferation, migration and invasion. Although HeLa cells may not represent the best model to test cancer behaviors due to the dysfunctional LKB1/AMPK signaling, a recently demonstrated pathway modulating NADPH homeostasis and promoting tumor cell survival during energy stress [38], our rescue experiments indicate that NAC prevents malignant phenotypes through inhibition of Notch3 signaling also in the LKB1/AMPK-proficient HCC1937 cells. Meanwhile, Notch3 silencing leads to similar depressive effects on these phenotypes, supporting that the malignant signaling is Notch3-dependent in these cells. Furthermore, the NAC-induced inhibition of Notch3 signaling and proliferation is observed in three additional cell lines (ZR-75-1, MCF-7 and OVCAR-3). These results together demonstrate that NAC negatively regulates Notch3 malignant signaling, and the regulation is not cell type-specific. On the other hand, dosages of NAC may determine how it functions in carcinogenesis. For example, it has been reported that NAC at a concentration of 0.2 mM increases malignant phenotypes in melanoma cell lines [39] and at 1 mM increases proliferation of lung cancer cells expressing wild-type p53 such as A549 and H460 [25]. In particular, A549 cells are considered to be Notch3-dependent. Nonetheless, these observations using low doses and ours using high doses are not mutually exclusive because NAC has no impact on Notch3 levels at 2 mM but displays an inhibitory role in malignancy at 5-10 mM. Such doses (5-10 mM) are attainable in vivo, as similar concentrations of NAC have been intravenously administered in animals [40–42]. However, clinical studies have shown that plasma concentrations of NAC range 300-900 mg/L, which equals 1.8-5.5 mM [43, 44]. Thus, NAC at a dose of 5 mM is clinically more relevant than 10 mM. Meanwhile, a role of ROS in tumorigenesis remains exist. Thus, further investigations are needed to elucidate how pathways associated with diminished ROS and the ROS-independent NAC-Notch3 pathway interplay in the regulation of malignancy.

In summary, our data demonstrate that NAC decreases Notch3 levels, but not Notch1, through a lysosome-mediated degradation pathway potentially by its interaction with the non-covalent binding region of Notch3 receptor. Such effect of NAC is independent of ROS. NAC suppresses the malignant phenotypes including proliferation, migration and invasion through inhibition of Notch3 signaling in cancer cells. Since NAC as a ROS scavenger is presumably cancer preventive, at least when functioning at the site of production [26], future studies are warranted to understand whether Notch3-expressing cancer cells display increased susceptibility to NAC treatment in vivo. Overall, our results provide mechanistic insight into a novel suppression of Notch3 and its malignant signaling by NAC in cancer cells, suggesting a promising application of NAC targeting Notch3-mediated carcinogenesis.

## MATERIALS AND METHODS

### Cells and materials

Human cell lines HeLa, ZR-75-1 and MCF-7 were obtained from American Type Culture Collection (Manassas, VA, USA). HCC1937 and OVCAR-3 cells were from the Cell Bank of Chinese Academy of Sciences (Shanghai, China). HeLa and MCF-7 cells were cultured in DMEM and the other cells were in RPMI-1640 media supplemented with 10% fetal bovine serum (Invitrogen, Carlsbad, CA, USA), 100 U/ml penicillin and 100 μg/ml streptomycin (Solarbio Science and Technology; Beijing, China). NAC, SOD and catalase were purchased from Beyotime (Shanghai, China). The γ-secretase inhibitor *N*-[*N*-(3,5-difluorophenacetyl)-L-alanyl]-*S*-phenylglycine *t*-butyl ester (DAPT) was from Calbiochem (San Diego, CA, USA). Ammonium chloride (NH_4_Cl) and lactacystin were from Sigma (St. Louis, MO, USA). Buthionine sulfoximine (BSO) were from Santa Cruz Biotechnology (Santa Cruz, CA, USA). Cytosolic, nuclear, and membrane fractions were extracted by using kits from Beyotime followingthe manufacturer's instructions.

### Western blot analysis

Cells were lysed in a sample buffer containing 2% SDS, 60 mM Tris-HCl (pH 6.8) and 5% glycerol. Cell lysates were then boiled for 5 min. Total protein concentration was determined using a BCA kit (Beyotime, Shanghai, China), and equal amount of protein was loaded for Western blot analysis. In brief, proteins were resolved by SDS-PAGE, transferred to a PVDF membrane (Pall Gelman Laboratory, MI, USA), and then blocked with 5% milk in TTBS for 1 h at room temperature. The membrane was then incubated overnight at 4°C with a primary antibody followed by a secondary horseradish peroxidase-conjugated anti-rabbit or anti-mouse antibody (Cell Signaling, Boston, MA, USA) for 1 h at room temperature. We used rabbit anti-Notch3 (sc-5593) and anti-Notch1 (sc-6014-R) antibodies (Santa Cruz Biotechnology, Santa Cruz, CA, USA) that target the C-terminus; therefore, the intracellular fragments and full length Notch precursor can be detected. Mouse anti-Notch3 NECD antibody (H00004854-M01, Novus Biologicals, Littleton, CO, USA) recognizes the extracellular fragments and full length Notch3 precursor. Rabbit anti-Hes1 and anti-HRT1 antibodies were from Millipore (Temecula, CA, USA). Rabbit anti-cyclin B1 and anti-Na^+^, K^+^-ATPase and mouse anti-β-actin antibodies were from Cell Signaling. Mouse anti-GAPDH antibody was from Beyotime. Blots were developed using enhanced chemiluminescence reagents (LumiGLO^®^Reagent and Peroxide, Cell Signaling) according to the manufacturer's instructions.

### Quantitative real-time PCR analysis

Total RNA was extracted from HeLa cells stored in TRIzol (Invitrogen, Carlsbad, CA, USA) and reverse-transcribed using the PrimeScript RTMaster Mix Perfect Real Time kit (TaKaRa, Dalian, China) following the manufacturers' instructions. Real-time PCR analyses of Hes1, HRT1 and Notch3 mRNA levels were performed in Mastercycler^®^ Realplex (Eppendorf, Germany) in sterilized 96-well PCR plates. The forward and reverse primer pairs were as follows: Hes1, 5′-GTCAACACGACACCGGATAA-3′ and 5′-GAGGTGCTTCACTGTCATTTCC-3′; HRT1, 5′-TGACCGTGGATCACCTGAAA-3′ and 5′-GCTGG GAAGCGTAGTTGTTG-3′; Notch3, 5′-GTGTGTGTC AATGGCTGGAC-3′ and 5′-GTGACACAGGAGGCC AGTCT-3′; β-actin, 5′-CGCGGCGGCGCCCTATAAA A-3′ and 5′-TGCACATGCCGGAGCCGTTG-3′. A volume of 20 μl mixture was used for reaction, which contained 37.5 ng cDNA, 300 nM primers and 10 μl SsoFast™ EvaGreen Supermix (Bio-Rad, Richmond, CA, USA). PCR reactions were performed at the following conditions sequentially: 10 sec at 95°C, 40 cycles of 95°C for 5 sec and 55°C for 30 sec, melting curve analysis (95°C for 15 sec, 60°C for 15 sec, from 60 to 95°C for 20 min, 95°C for 15 sec), and then hold at 4°C.

### Vector and siRNA transfection

The vectors expressing full-length Notch3, N3ICD, or Hes1 firefly luciferase reporters [33, 45] were generously provided by Dr. Michael Wang, University of Michigan, USA. The Renilla luciferase vector pRL-TK was from Promega (Madison, WI, USA). Scrambled and Notch3 siRNA sequences (5′-CCUGGCUACAAUGGUGAUATT-3′) were purchased from GenePharma (Shanghai, China). Vectors or siRNA sequences were transfected into cells using Lipofectamine 2000 (Invitrogen). For dual-luciferase reporter assay, cells were co-transfected with the Hes1 reporter (200 ng) and pRL-TK (10 ng) vectors for 12 h. After overnight serum starvation, the cells were treated with NAC for 12 h. Luciferase activity was measured using the dual-luciferase reporter assay kit (Promega) following the manufacturer's instructions.

### Measurement of intracellular ROS

Production of ROS was fluorometrically monitored using 2′,7′-dichlorofluorescein diacetate (DCFH-DA, Beyotime, Shanghai, China). Briefly, HeLa cells treated with catalase (1200 U/ml) or NAC (5 mM) for 0, 3, 6 or 12 h were incubated with DCFH-DA (10 μM in fresh DMEM) for 20 min at 37°C, trypsinized, washed three times with PBS, and then loaded for reading at 488 nm (excitation) and 525 nm (emission) using FACSCalibur flow cytometer (BD Biosciences, USA).

### Measurement of total GSH

Total GSH content (reduced and oxidized forms) was measured using a kit from Beyotime (Shanghai, China) following the manufacturer's instructions. In brief, cell homogenates were deproteinated and the supernatant was processed to measure total GSH content with the reagents 5,5-dithio-bis-(2-nitrobenzoic acid), glutathione reductase and NADPH. The color formation was spectrophotometrically determined at 412 nm.

### Proliferation assay

Cells were seeded in 12-well plates and allowed to grow for 24 h prior to plasmid or siRNA transfection. Twenty-four hours after transfection with the N3ICD or empty vector, cells were treated with NAC (0, 5 or 10 mM) and collected at 12, 24 and 48 h. Alternatively, cells were trypsinized at 24, 48 and 72 h after siRNA delivery. Viable cells were counted based on the Trypan blue exclusion method [10].

### Wound healing assay

Wound healing assays were performed as described by Song G et al [46]. Briefly, cells were seeded in 12-well plates for overnight and transfected with the vector or siRNA sequence. Cells were grown to confluence 36 h post-transfection and wounded by dragging a 200-μl pipette tip through the monolayer. Cells were then washed using pre-warmed PBS to remove cellular debris and allowed to migrate for 24 h. Cells containing the N3ICD-expressing or empty vector were treated with NAC (0, 5 or 10 mM) at the time of wounding. Images of cell migration were taken at 0 and 24 h after wounding under a microscope (ECLIPSE Ti, Nikon, Japan). The relative distance between the leading edges was determined using NIS-Elements Documentation Software 4.10 and expressed as a migration index (the distance migrated in 24 h relative to the initial scraped gap).

### Invasion assay

Cell invasion was analyzed as described previously with minor modifications [47]. Matrigel (BD Biosciences, Franklin Lakes, NJ, USA) was thawed at 4°C overnight and diluted to 3 mg/ml with cold DMEM. Millicell^®^ plate inserts (8.0 μm pore size, 24-well format, BD Biosciences) were coated with 50 μl diluted Matrigel and incubated at 37°C for 4-5 h to allow for gelling. Cells were starved overnight 24 h after being transfected with the vectors or siRNA sequences. Cells containing the N3ICD or empty vector were treated with NAC (0, 5 or 10 mM) for 6 h, trypsinized, washed twice with DMEM, and resuspended in DMEM at a density of 10^6^ cells/mL. Lower chambers were filled with 500 μl warm DMEM containing 10% fetal bovine serum as the attractant, while the Matrigel-coated upper chambers were filled with 200 μl of the cell suspension. Following 24 h incubation at 37°C, cells were fixed for 30 min in 5% glutaraldehyde prepared in PBS at room temperature, and stained for 30 min in 0.1% crystal violet dissolved in 2% ethanol. The numbers of invasive cells were calculated by counting three fields per chamber.

### Statistics

Statistical analyses of the data were performed by Student's *t*-test for paired observations. Data are expressed as means ±SE from at least three independent experiments. Differences were considered statistically significant when *p* < 0.05.

## SUPPLEMENTARY FIGURES


