# Supplementary material for: N-acetylcysteine negatively regulates Notch3 and its malignant signaling
Source: Oncotarget. 2016 Apr 18;7(21):30855–66. doi: 10.18632/oncotarget.8806 (PMC5058723; doi:10.18632/oncotarget.8806)
Supplement: Supplementary file 1 [file oncotarget-07-30855-s001.pdf]

SUPPLEMENTARY FIGURES

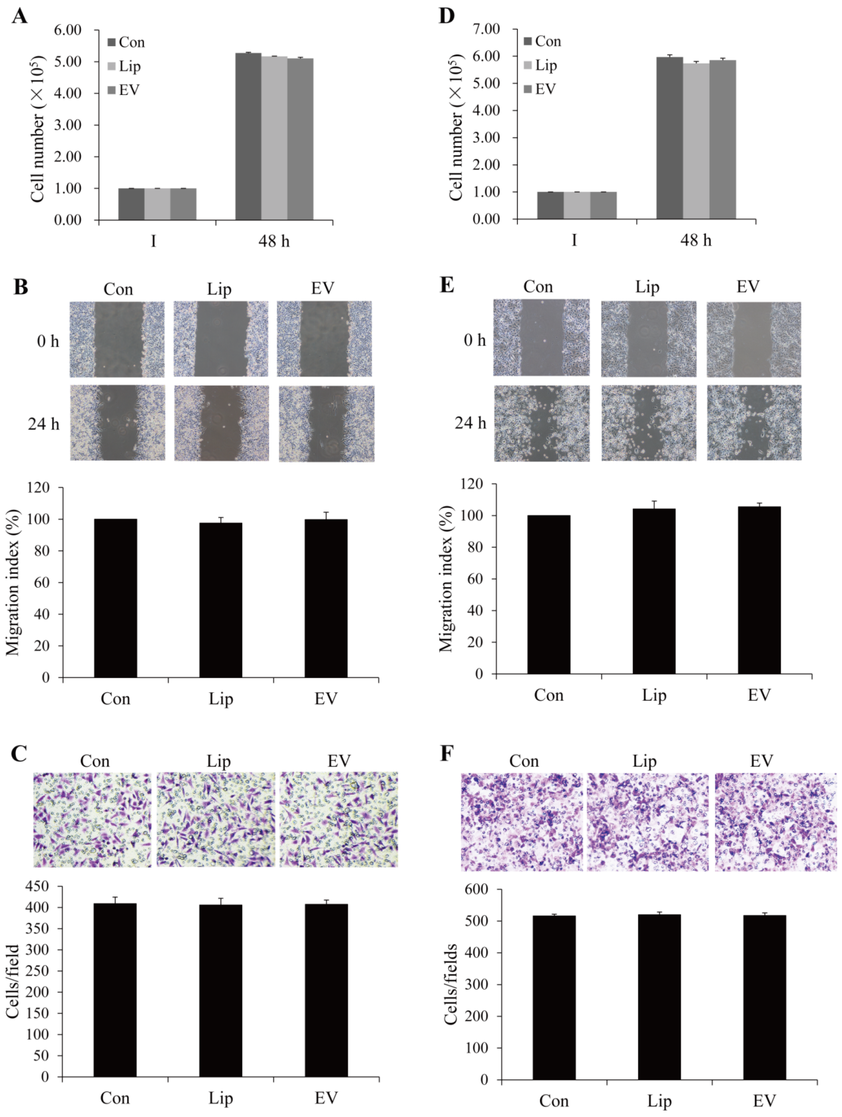

Supplementary Figure S1: Comparison of cells of naive (Con), Lipofectamine 2000 only (Lip) and empty vector-transfected (EV) in proliferation, migration and invasion. Cells were treated with 5 mM NAC. Representative images were shown for migration and invasion. **A-C.** Comparison of HeLa cells in proliferation (A), migration (B) and invasion (C). **D-F.** Comparison of HCC1937 cells in proliferation (D), migration (E) and invasion (F). Cell numbers or cells per field on the insert membrane were counted for proliferation and invasion, respectively. Results for migration were expressed as the migration index, that is, the distance migrated relative to the initial scraped gap, and the one of naive cells was set as 100%. Data are presented as mean ± SE, n = 3.

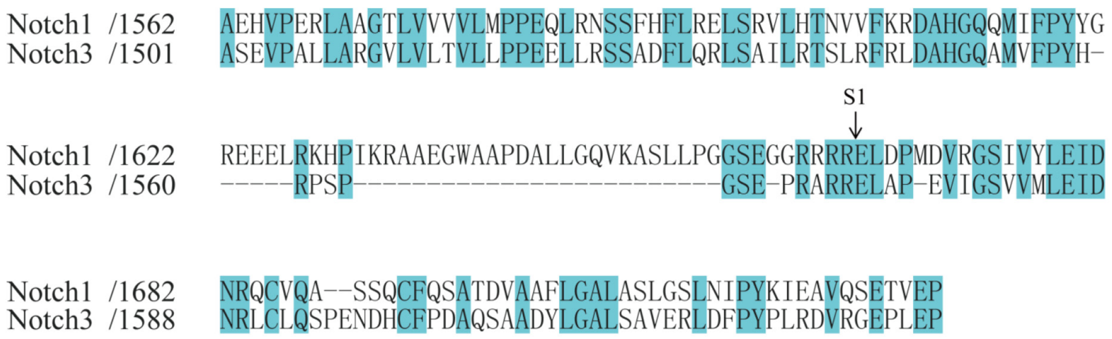

Supplementary Figure S2: Sequence alignment of the dimerization regions of Notch1 and Notch3.
